# The Role of Replication Clamp-Loader Protein HolC of Escherichia coli in Overcoming Replication/Transcription Conflicts

**DOI:** 10.1128/mBio.00184-21

**Published:** 2021-03-09

**Authors:** Deani L. Cooper, Taku Harada, Samia Tamazi, Alexander E. Ferrazzoli, Susan T. Lovett

**Affiliations:** a Department of Biology, Rosenstiel Basic Medical Sciences Research Center, Brandeis University, Waltham, Massachusetts, USA; Vanderbilt University.

**Keywords:** DNA repair, DNA replication, stringent response, transcription factors

## Abstract

In Escherichia coli, DNA replication is catalyzed by an assembly of proteins, the DNA polymerase III holoenzyme. This complex includes the polymerase and proofreading subunits, the processivity clamp, and clamp loader complex. The *holC* gene encodes an accessory protein (known as χ) to the core clamp loader complex and is the only protein of the holoenzyme that binds to single-strand DNA binding protein, SSB. HolC is not essential for viability, although mutants show growth impairment, genetic instability, and sensitivity to DNA damaging agents. In this study, we isolate spontaneous suppressor mutants in a Δ*holC* strain and identify these by whole-genome sequencing. Some suppressors are alleles of RNA polymerase, suggesting that transcription is problematic for *holC* mutant strains, or alleles of *sspA*, encoding stringent starvation protein. Using a conditional *holC* plasmid, we examine factors affecting transcription elongation and termination for synergistic or suppressive effects on *holC* mutant phenotypes. Alleles of RpoA (α), RpoB (β), and RpoC (β′) RNA polymerase holoenzyme can partially suppress loss of HolC. In contrast, mutations in transcription factors DksA and NusA enhanced the inviability of *holC* mutants. HolC mutants showed enhanced sensitivity to bicyclomycin, a specific inhibitor of Rho-dependent termination. Bicyclomycin also reverses suppression of *holC* by *rpoA*, *rpoC*, and *sspA*. An inversion of the highly expressed *rrnA* operon exacerbates the growth defects of *holC* mutants. We propose that transcription complexes block replication in *holC* mutants and that Rho-dependent transcriptional termination and DksA function are particularly important to sustain viability and chromosome integrity.

## Abstract (precis)

Transcription elongation complexes present an impediment to DNA replication. We provide evidence that one component of the replication clamp loader complex, HolC, of Escherichia coli is required to overcome these blocks.

## INTRODUCTION

The ability to replicate DNA faithfully is critical for the survival of all organisms. The replication fork very frequently encounters barriers that need to be overcome to ensure cell survival and genetic stability (1, 2). Such barriers may be breaks, nicks, or modified bases in the DNA template, damage to the deoxynucleoside triphosphate (dNTP) pool or nascent strand, tightly bound proteins, transcription complexes, and DNA secondary structures. Single-stranded gaps left behind by the fork can be filled by a number of mechanisms found broadly across organisms, including homologous recombination with the sister chromosome, translesion DNA synthesis, and template switching (3).

The bulk of DNA replication in the bacterium Escherichia coli is catalyzed by the DNA polymerase III holoenzyme (4, 5). This multisubunit complex consists of the core DNA polymerase assemblage with a proofreading exonuclease subunit (α, ε, φ), the processivity clamp (β), and an associated clamp-loader complex ([τ/γ]_3_δδ′) with its accessory complex (χψ). (Because the same Greek letters are used for subunits of DNA polymerase [Pol] III and RNA polymerase [RNAP], for simplicity, we use gene names here to designate the DNA Pol III proteins and Greek letters for RNAP.) The processivity clamp is a ring-like structure that encircles DNA and tethers DNA polymerases to their templates, conferring processivity to DNA synthesis. The pentameric clamp loader complex can both load and unload the clamp, a cycle that must be completed each round of Okazaki fragment synthesis on the lagging strand. The structures of the clamp and the clamp loader are conserved in all domains of life; in archaea and in eukaryotes, they are known as PCNA (proliferating nuclear antigen) and RFC (replication factor C), respectively. In E. coli, the clamp binds all of its 5 DNA polymerases (6); in addition to DNA Pol III, it binds Pol I, involved in Okazaki fragment maturation and RNA primer processing (7), and the DNA repair polymerases II, IV, and V (8).

Most of the proteins in the DNA polymerase III holoenzyme are essential for viability with some notable exceptions, two of which are HolC and HolD (or χ and φ, respectively) that form an accessory heterodimer that binds to the core pentameric clamp loader complex. HolC and HolD are not ubiquitous in bacteria and are found only in gammaproteobacteria, although there may be more unrelated proteins that play similar roles in other bacteria. HolC is of particular interest because it is the only protein of the DNA Pol III holoenzyme that binds single-strand DNA binding protein, SSB, at a site distinct from its interaction with HolD (9). At the opposite face of its interaction site with HolC, HolD interacts with the DnaX-encoded subunits of the pentameric clamp loader (10). Therefore, together, HolC and HolD form a bridge between SSB-coated template DNA, the pentameric clamp loader complex, and the rest of the DNA Pol III holoenzyme.

*In vitro* studies have suggested a number of roles for the HolC/D accessory complex in DNA replication. There is evidence that the HolC/D complex assists assembly and stability of the clamp loader complex (11) and increases its efficiency of clamp loading (12). HolC, through its interaction with SSB, aids the engagement of DNA Pol III with RNA primers and generally stabilizes interaction of the replisome with its template (9, 13, 14). HolD, through its interaction with DnaX proteins, induces higher affinity of the clamp loader for the clamp and for DNA (15, 16).

Deletion mutants of HolC are viable but grow quite poorly, and their cultures rapidly develop genetic suppressor variants. HolC mutants, even when grown under conditions that ameliorate their inviability, exhibit elevated rates of local genetic rearrangements, as do many mutants with other impairments in the DNA replication machinery (17). Mutants of HolC lacking its interaction with SSB cause temperature-dependent induction of the SOS DNA damage response and cell filamentation, with a block to chromosome partitioning (9). All in all, these phenotypes point to the aberrant nature of replication in the absence of HolC function.

Michel and collaborators have reported several studies of suppressor mutations that improve the viability of strains that lack HolC’s partner, HolD. A duplication of the *ssb* gene is one such suppressor, which suppresses loss of either HolC or HolD or both (18). This suggests that single-stranded DNA (ssDNA) gaps accumulate in HolCD mutant strains; extra SSB may protect ssDNA and recruit repair factors (19) to aid gap filling. Accumulation of ssDNA induces E. coli’s DNA damage response, the “SOS” pathway; blocking this with a noninducible allele of the SOS repressor, LexA [LexA(Ind^−^)], also improves the viability of HolD mutants (20). The negative effect of the SOS response in HolD mutant strains is due to increased expression of the translesion DNA polymerases, DNA polymerase II and DinB (20) and, to a lesser extent, to a SulA-dependent block to cell division. Mutations in the replisome-associated ATPase RarA (21), implicated in DNA polymerase exchange (22), are also partial suppressors, and its suppression of HolD is epistatic to LexA(Ind^−^) (23), indicating a common mechanism. These results suggest that the accumulation of replication gaps in HolD mutants trigger the SOS response, including the upregulation of translesion DNA polymerases Pol II and Pol IV, which compete with DNA Pol III, replacing it on the clamp. Because these polymerases are slower or more error prone than Pol III (24, 25), this polymerase exchange may be deleterious. An L32V allele of the clamp-loader subunit, DnaX, to which HolD binds, was also found as a suppressor (23) and may increase the stability or functionality of the clamp loader complex in the absence of HolD. Likewise, mutations affecting K^+^ import, TrkA, and RfaP may also suppress HolD by this mechanism (26). Finally, inactivation of the stringent starvation protein SspA suppresses HolD by an unknown mechanism, genetically distinct from SOS, RarA, and TrkA (23).

It had been assumed that the function of HolC and HolD are obligately linked. However, HolC is implicated in repair of damaged forks in a way that HolD is not. HolC physically interacts with a putative DNA helicase of the XP-D/DinG family, YoaA, that is induced by DNA damage; both enhance survival to the replication chain terminator nucleoside 3′ azidothymidine, AZT (27), that produces gaps during replication (28). Recently, we have provided evidence that the HolC YoaA and HolC HolD complexes are mutually exclusive (29). Both HolD and YoaA appear to bind to the same surface of HolC, including residues W57 and P64, at a site distal to the residues required for interaction with SSB (10). We proposed that, after DNA damage and the accumulation of unreplicated DNA, this mechanism allows the recruitment of the YoaA helicase to the fork, without accompanying DNA Pol III molecules.

To clarify the role of HolC in replication and repair, we characterize here a number of spontaneous suppression mutations to *holC*.

## RESULTS

A Δ*holC* strain was grown overnight in minimal medium at 30°C, conditions that minimize toxicity, and plated on LB or LB with AZT and incubated at 37°C overnight. Under these conditions, Δ*holC* strains grow poorly and form small colonies. We isolated large colony variants, which were purified to single colonies on minimal medium at 30°C. Because AZT must be phosphorylated before it can be incorporated into DNA, many spontaneous AZT-resistant derivatives have mutations in the *tdk* gene, encoding thymidine kinase (30) of the thymidine salvage pathway, and often are deletions of all or part of the locus. We used a colony PCR assay to screen these out. Twenty-six strains, 11 derived from selection on LB and 15 from selection on LB-AZT, with a wild-type (wt)-length *tdk* locus were frozen, and DNA was prepared for whole-genome sequencing. Of the 15 AZT-resistant isolates, 6 had point mutations in the *tdk* gene and were not pursued further. Among the remaining strains, several piqued our interest: 3 isolates had mutations in RNA polymerase {RpoA R191C, RpoA duplication of amino acids 179 to 186 [dup(aa179-186)], and RpoC E756K}, one had an alteration in the replication fork helicase (DnaB E360V), and 2 had mutations in stringent starvation protein SspA (SspA Y186S and A to C in its upstream Shine-Dalgarno sequence). All of these except RpoA R191C were isolated as faster-growing variants on LB; RpoA R191C was selected as an AZT-resistant isolate.

To study the genetic properties of these suppressors in the absence of selection for growth, we engineered a *holC* conditional mutant, with *holC* deleted on the chromosome and a plasmid encoding *holC*^+^ (pAM34-*holC*) that can be retained only in the presence of isopropyl-β-d-thiogalactopyranoside (IPTG). In medium with IPTG, cells are HolC^+^; without IPTG, the complementing plasmid is lost and the *holC* mutant phenotype is revealed.

**FIG 1:**
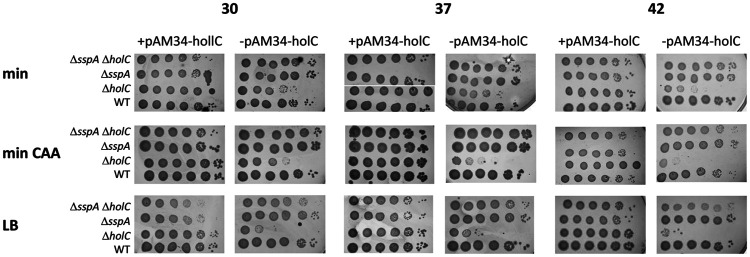
Suppression of *holC* by *sspA*. Tenfold serial dilutions of cultures with and without the *holC* complementing plasmid (+pAM34-*holC* and −pAM34-*holC*, respectively) were plated on minimal glucose (min), minimal glucose Casamino Acids (min CAA), or LB medium and incubated at 30°C, 37°C, and 42°C, as indicated.

**FIG 2:**
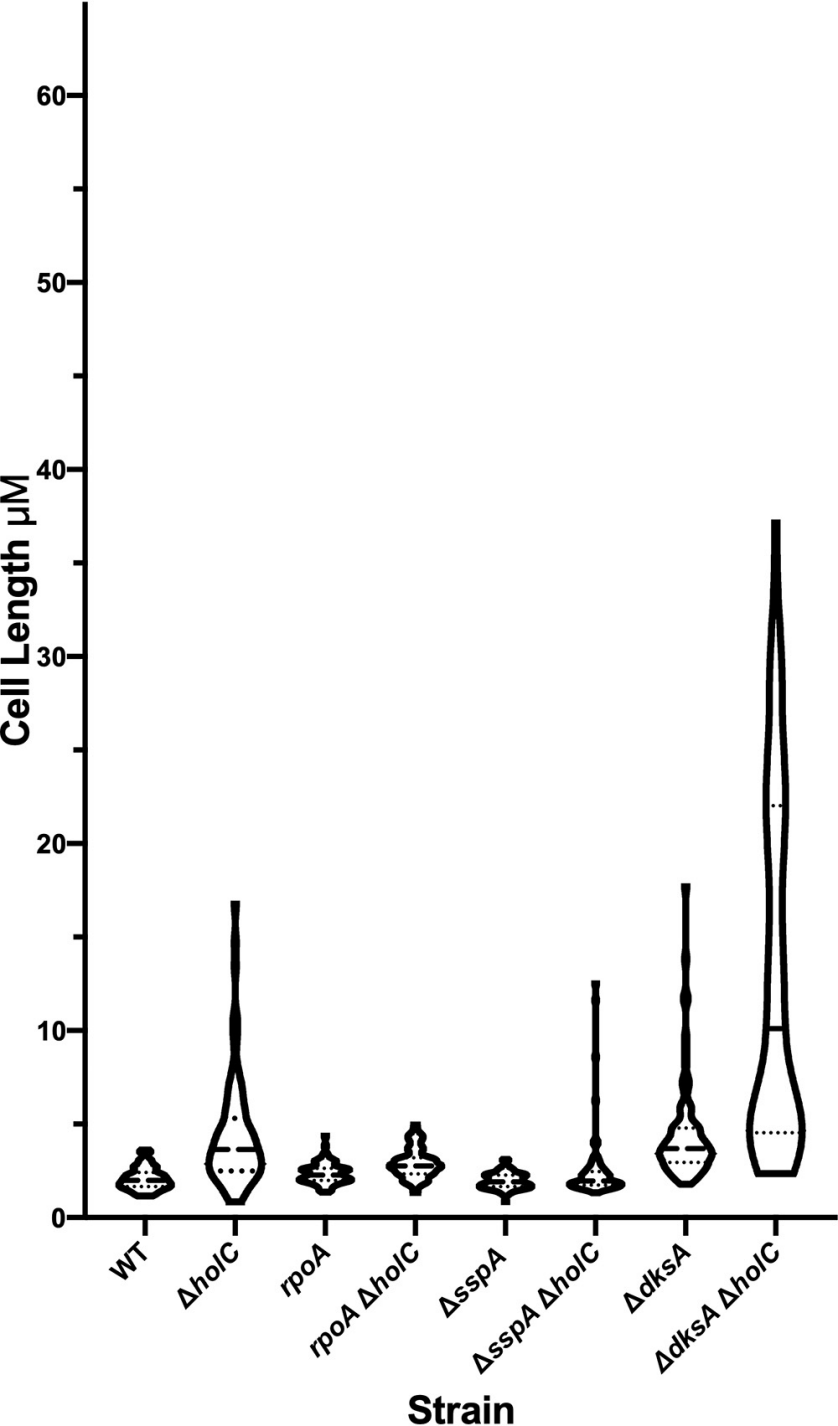
Violin plot of the cell length distribution of *holC* mutant derivatives grown in min CAA at 30°C as determined by microscopy. The dashed lines indicate the median values and dotted lines the quartile values.

In a study by Michel and Sinha (23), loss-of-function mutations in *sspA*, encoding a transcriptional activator protein, were found to suppress *holD*. We recovered two alleles of *sspA* among our suppressed *holC* strains. Because it is not clear what effects our alleles would have on *sspA* function, rather than characterizing them further, we assayed the consequence of an *sspA* knockout mutation on *holC* phenotypes in the conditional strain. Growth defects of *holC* mutants (lacking the pAM34-*holC* plasmid) were enhanced with richer growth medium (LB > minimal glucose Casamino Acids [min CAA] > minimal glucose [min]) and at higher temperatures. Concomitant loss of *sspA* function provided full suppression of *holC* growth defects under all conditions (Fig. 1). Suppression of *holC* by *sspA* was most dramatic under the most restrictive condition, LB at 42°C, where plating efficiency was increased by 4 orders of magnitude. Mutants in *holC* cured of the complementing plasmid showed a broad distribution of increased cell lengths (Fig. 2), including long cell filaments; addition of *sspA* largely returned this distribution to one similar to wt.

**FIG 3:**
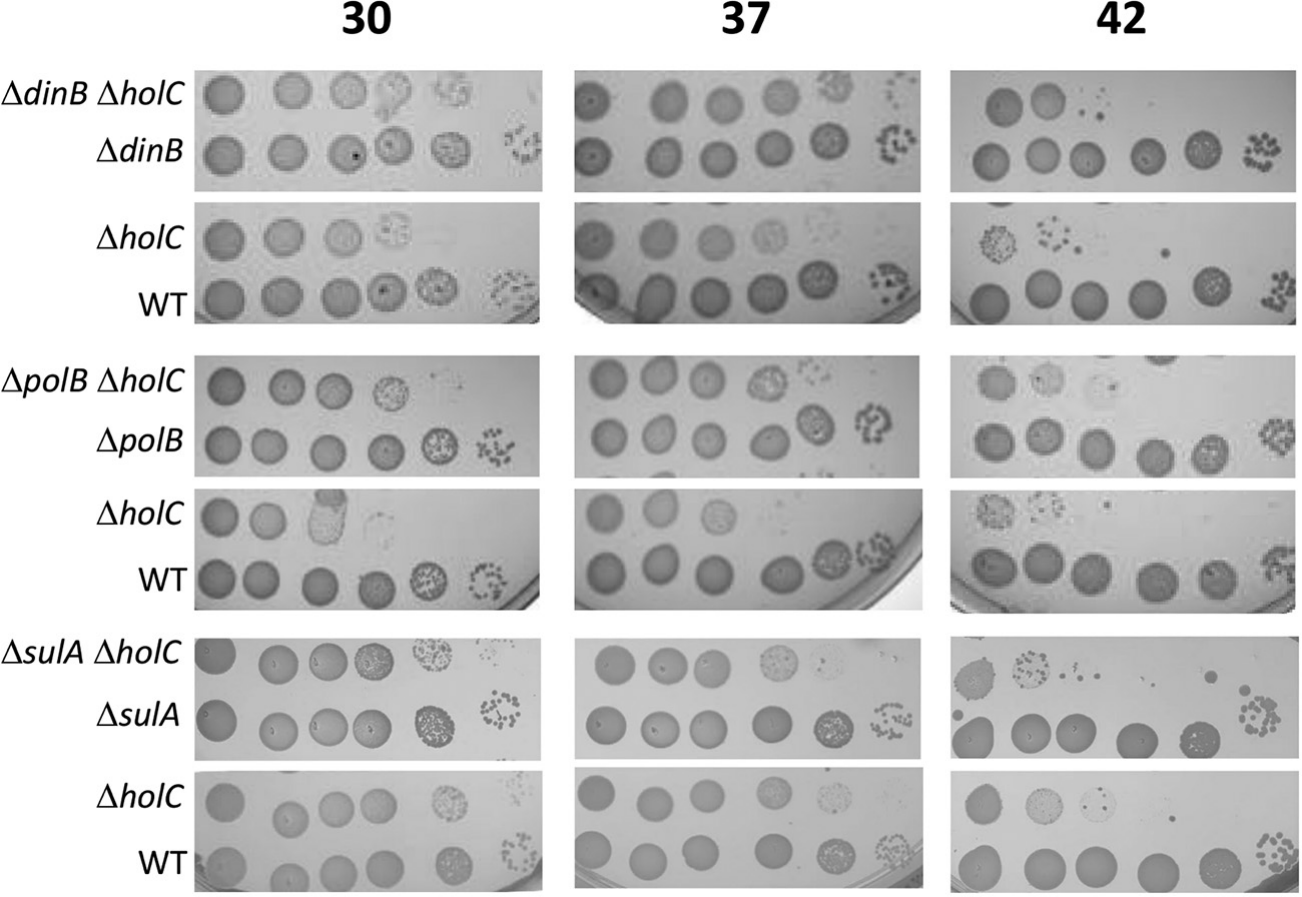
Suppression of *holC* by SOS-induced functions. Tenfold serial dilutions of cultures cured for the *holC* complementing plasmid (pAM34-*holC*) were plated on minimal glucose (min) medium and incubated at 30°C, 37°C, and 42°C, as indicated.

Michel et al. also found that in *holD* mutants, RecF-dependent induction of the SOS response contributes to its poor growth phenotype (20). Some toxicity conferred by *holD* was relieved by inactivation of SOS-induced DNA polymerases Pol II (*polB*) and Pol IV (*dinB*), implicating polymerase exchange as a contributor to toxicity in *holD* strains. We likewise found a modest increase in plating efficiency of the *holC* mutants in strains lacking *polB* or *dinB* (Fig. 3) (suppression was most evident at 30°C and 37°C). As observed for *holD* mutants (20), we saw little or no suppression of the plating defects of *holC* mutants by *sulA*, the cell division inhibitor induced by the SOS response.

**FIG 4:**
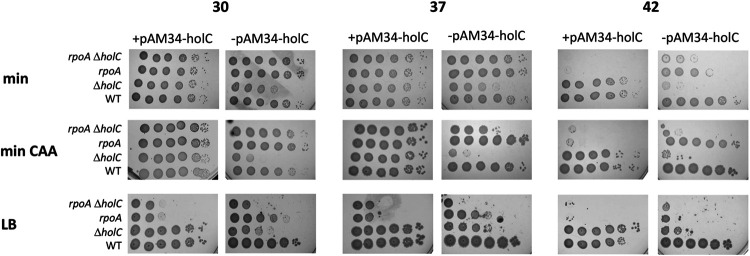
Suppression of *holC* by *rpoA* dup(aa179-186). Tenfold serial dilutions of cultures with and without the *holC* complementing plasmid (+pAM34-*holC* and −pAM34-*holC*, respectively) were plated on minimal glucose (min), minimal glucose Casamino Acids (min CAA), or LB medium and incubated at 30°C, 37°C, and 42°C, as indicated.

Most intriguing were the suppressor isolates affecting RNA polymerase (RNAP), which were not identified in prior studies of *holD* suppression. By genetic backcrosses, we showed that the RpoA duplication of amino acids 179 to 186 was sufficient to suppress the poor growth of *holC* mutants under many conditions (Fig. 4). Growth of the *holC* mutant in the absence of IPTG was poor, especially on rich medium and at higher temperatures. The suppression by RpoA dup(aa179-186) of *holC* mutants was not complete, and some inviability was retained at higher temperatures and on LB (Fig. 4). However, the RpoA dup(aa179-186) single mutant strain itself was LB sensitive and temperature sensitive. In addition, the *holC*^+^ plasmid (a ColEI medium-copy-number derivative) appeared to be toxic to *rpoA* mutants, especially on LB and at higher temperature: survival was enhanced after plasmid loss (right panels versus left panels, Fig. 4). Filamentation to larger cell length in *holC* strains was also ameliorated by RpoA dup(aa179-186) (Fig. 2).

The other *rpoA* allele isolated in the screen, R191C, is the same mutation as *rpoA101*, a well-characterized temperature-sensitive allele of RNAP α (31, 32); RNAP assembles with normal kinetics in this mutant but is unstable, with β and β′ rapidly degraded. Because its intrinsic temperature sensitivity would confound that of *holC*, we did not characterize this allele further.

**FIG 5:**
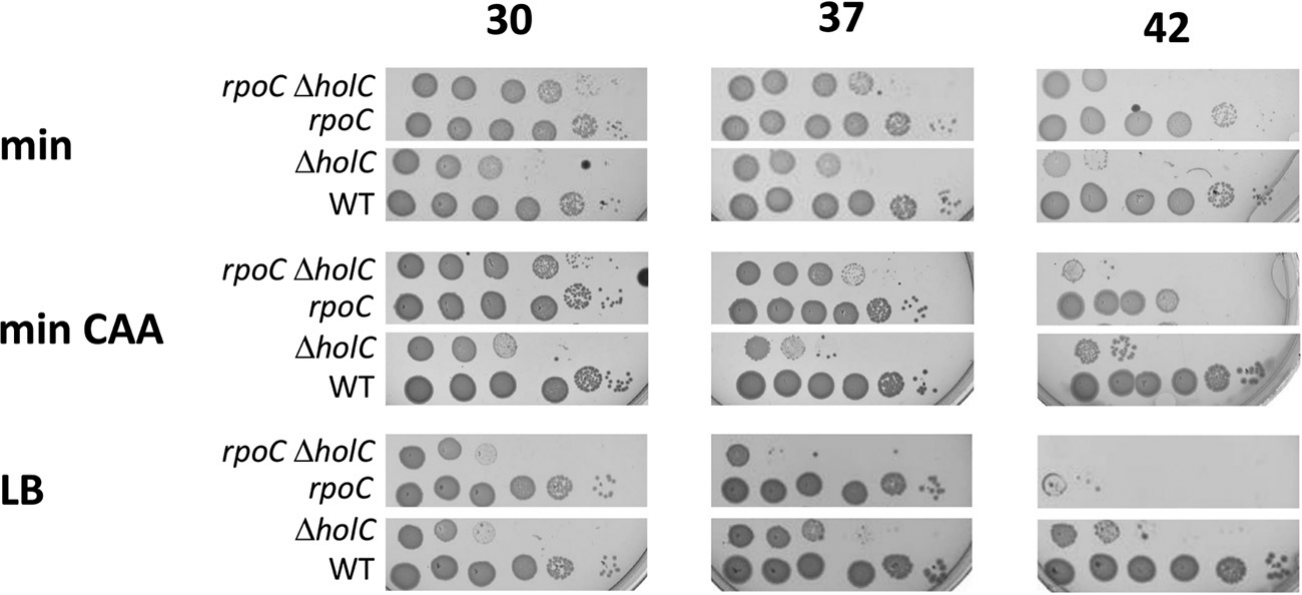
Suppression of *holC* by *rpoC*::GFP. Tenfold serial dilutions of cultures without the *holC* complementing plasmid were plated on minimal glucose (min), minimal glucose Casamino Acids (min CAA), or LB medium and incubated at 30°C, 37°C, and 42°C, as indicated.

We were unable to recover the *rpoC* E756K strain, but during the course of genetic analysis, we discovered that an *rpoC*::green fluorescent protein (GFP) fusion allele was able to partially suppress the *holC* phenotypes (Fig. 5). This allele was considered to be functional but somewhat temperature sensitive (33), although it can sustain viability in the absence of other *rpoC* genes at lower temperatures. This suppressive effect confirms that it must be perturbed in some way. Like *rpoB* dup(aa179-186) *holC*^+^ strains, *rpoC*::GFP *holC*^+^ strains were also LB sensitive, especially at high temperature.

Transcription complexes pose a major impediment to the replication fork (34–36). To decipher the mechanism of RNAP suppression of a DNA replication mutant, we examined mutants in several factors known to modulate transcription elongation for their effects, positive or negative, on *holC* mutant phenotypes. GreA and GreB are elongation factors that reactivate backtracked transcription elongation complexes by promoting cleavage of the RNA 3′ terminus to reposition it in the active center of the enzyme (37, 38). Neither of these functions are essential for viability and neither had effects on *holC* phenotypes (see Fig. S1 in the supplemental material and data not shown). Mfd mediates transcription-coupled repair, where excision repair proteins are recruited to sites of RNAP arrest (39). In addition, through its ATP-dependent translocase activity, Mfd promotes RNAP release from the DNA template (40, 41). Loss of Mfd neither enhanced nor suppressed *holC* inviability (Fig. S1).

10.1128/mBio.00184-21.1 FIG S1 Lack of effect on *holC* growth defects by *greA* and *mfd*. Tenfold serial dilutions of cultures with and without the *holC* complementing plasmid were plated on minimal glucose medium and incubated at 30°C, 37°C, and 42°C, as indicated. Download FIG S1, PDF file, 1.4 MB. Copyright © 2021 Cooper et al. 2021 Cooper et al. https://creativecommons.org/licenses/by/4.0/ This content is distributed under the terms of the Creative Commons Attribution 4.0 International license.

**FIG 6:**
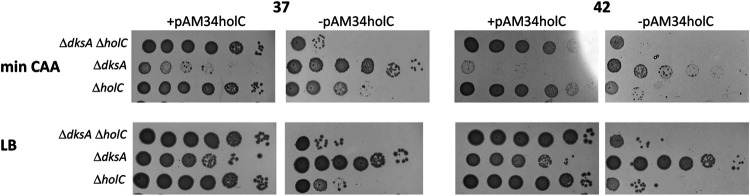
Enhancement of *holC* growth defects by *dksA*. Tenfold serial dilutions of cultures with and without the *holC* complementing plasmid (+pAM34-*holC* and −pAM34-*holC*, respectively) were plated on minimal glucose Casamino Acids (min CAA) or LB medium and incubated at 37°C and 42°C, as indicated.

**FIG 7:**
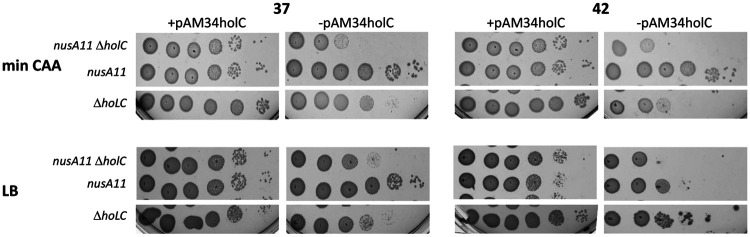
Enhancement of *holC* growth defects by *nusA11*. Tenfold serial dilutions of cultures with and without the *holC* complementing plasmid (+pAM34-*holC* and −pAM34-*holC*, respectively) were plated on minimal glucose Casamino Acids (min CAA) or LB medium and incubated at 37°C and 42°C, as indicated. These strains are derived from AB1157 (ΔRac) in which lethal effects of *nusA* mutations are reduced.

DksA is structurally similar to the Gre proteins and binds to the secondary channel of RNAP; it affects both the initiation and elongation properties of RNAP, especially in the presence of the signaling molecule ppGpp (reviewed in reference 42). *In vivo*, there is evidence that DksA alleviates conflicts between replication and transcription, preventing replication arrest by stalled transcription complexes during amino acid starvation (43). Mutants in *dksA* had synthetic growth defects when combined with *holC* (Fig. 6), indicating that DksA function protects replication in the absence of *holC*. Loss of *dksA* also exacerbated cell filamentation in *holC* mutants (Fig. 2). Surprisingly, the *holC* plasmid inhibited growth of the *dksA* (*holC*^+^) strain on minimal CAA medium; we do not know the basis of this effect. Like DksA, a mutant of NusA, *nusA11*, reduced the plating efficiency of *holC* mutants (Fig. 7). NusA potentiates Rho-dependent termination, which *in vivo*, prevents replication fork collapse and double-strand break formation (44).

**FIG 8:**
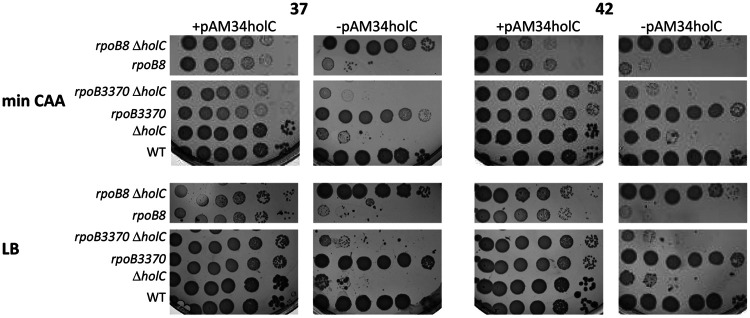
Suppression of *holC* growth defects by *rpoB8* and enhancement by *rpoB3370*. Tenfold serial dilutions of cultures with and without the *holC* complementing plasmid (+pAM34-*holC* and −pAM34-*holC*, respectively) were plated on minimal glucose Casamino Acids (min CAA) or LB medium and incubated at 37°C and 42°C, as indicated.

To further explore the role of transcriptional termination in the phenotypes of *holC* mutants, we examined a mutation in the β subunit of RNAP, *rpoB8* (Q513P), that increases transcriptional pausing, has a slower elongation speed, and is more prone to Rho-dependent termination (45–47). The *rpoB8* allele significantly suppressed *holC* inviability, indicating that Rho-dependent termination aids the viability of *holC* mutants (Fig. 8). Interestingly, suppression was mutual. The *holC* mutation also suppressed the poor growth of *rpoB8* strains on either min CAA or LB; the plasmidless *holC rpoB8* double mutant grew more robustly than either *holC* or *rpoB8* single mutants. We also examined effects of *rpoB3770* (T563P) that, like *rpoB8*, confers resistance to rifampicin. This is a “stringent” allele of *rpoB* that suppresses phenotypes of mutants defective in mounting the stringent response to starvation via accumulation of the signaling molecule (p)ppGpp (48). In contrast to *rpoB8*, *rpoB3370* did not ameliorate *holC* growth phenotypes and further reduced colony size upon loss of the *holC*^+^ complementing plasmid. This finding is consistent with a *holC*-suppressive effect of Rho-dependent termination, since strains carrying *rpoB3370* exhibit decreased termination at three different Rho-dependent terminators from bacteriophage lambda (49).

**FIG 9:**
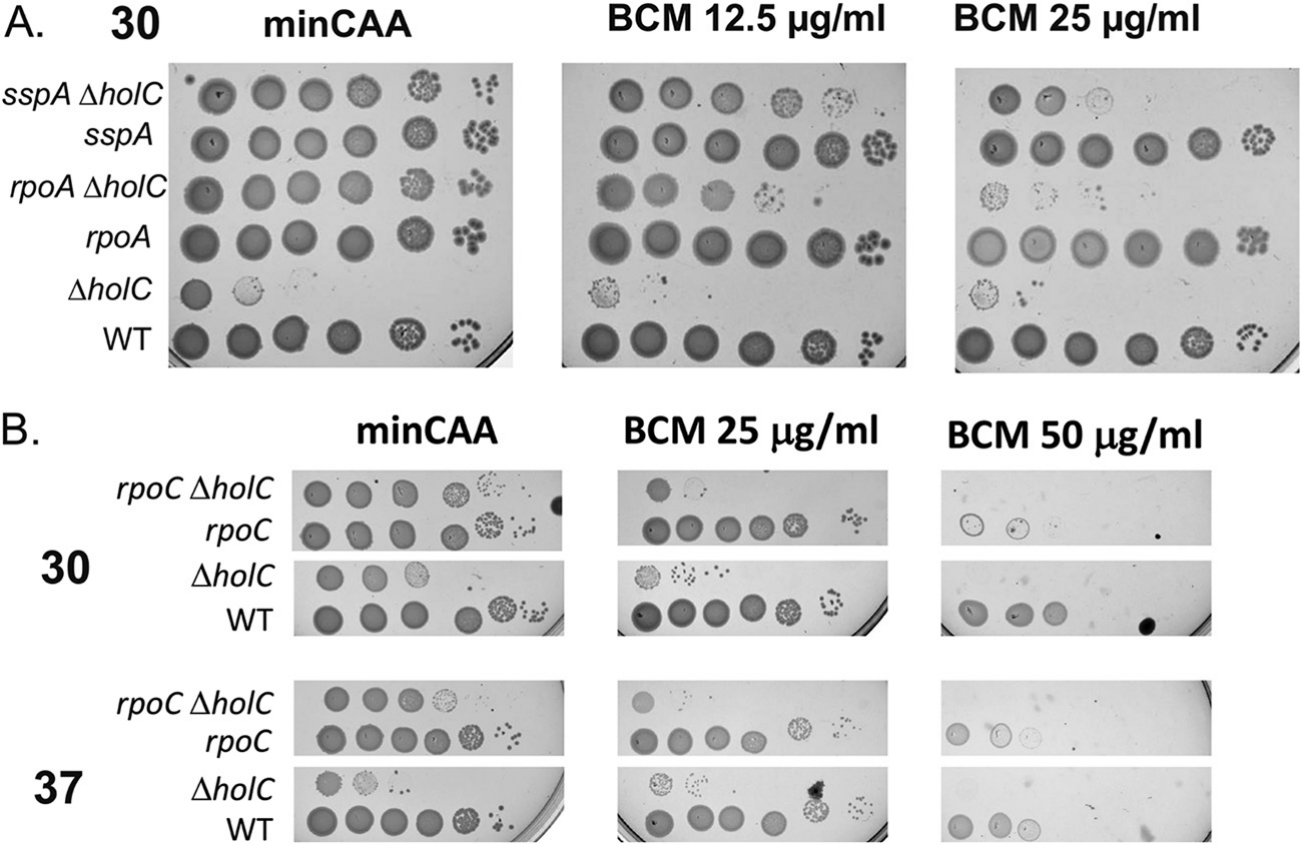
Bicyclomycin (BCM) sensitivity of *holC* mutants and *holC* suppression. Tenfold serial dilutions of cultures cured of the *holC* complementing plasmid were plated on minimal glucose Casamino Acids (min CAA) without and with two doses of bicyclomycin. (A) The *rpoA* allele is *rpoA* dup(aa179-186) and the *sspA* allele is a deletion. Suppression of *holC* by these alleles is reduced or abolished at 30°C on min CAA medium. (B) The *rpoC* allele is the *rpoC*::GFP allele, which partially suppresses *holC* on min CAA at 30°C and 37°C but not in the presence of bicyclomycin.

To determine the role of Rho-dependent termination in sustaining the viability of *holC* mutants, we tested the impact of bicyclomycin on phenotypes in *holC* and *holC*-suppressed strains. The antibiotic bicyclomycin is a specific inhibitor of Rho-dependent termination (50). Treatment of E. coli cells with bicyclomycin induces replication-dependent double-strand breaks of DNA, indicative of the collapse of replication forks (44). Moreover, mutations that weaken transcription elongation complexes partially suppress this effect, supporting the hypothesis that Rho displaces RNAP before or after its collision with the replisome (44). We found that *holC* mutants were abnormally sensitive to the killing effects of bicyclomycin, consistent with the notion that transcription/replication conflicts are more prevalent or more deleterious in the absence of HolC (Fig. 9A and B). The effect was seen at both 30°C and 37°C, where plating efficiency of the *holC* mutant was reduced approximately 10-fold by 25 μg/ml bicyclomycin (BCM), whereas that of the wild type was unchanged. Loss of *sspA* completely suppressed the *holC* mutant at 30°C on min CAA medium; suppression was reduced 2 orders of magnitude by BCM (Fig. 9A). Likewise, the *rpoA* dup(aa179-186) completely suppressed *holC* and suppression was abolished by BCM (Fig. 9A). Neither *sspA* nor *rpoA* dup(aa179-186) by itself promoted sensitivity to BCM (Fig. 9A). Suppression of *holC* by *rpoC* was also lost in the presence of bicyclomycin (Fig. 9B). This supports the notion that Rho-dependent termination, specifically inhibited by BCM, is required to sustain viability in the absence of *holC.* In addition, the ability of *rpoA*, *rpoC*, and *sspA* mutations to suppress *holC* are all dependent on Rho-dependent termination, indicating that these suppressor alleles may act through effects on Rho-dependent transcriptional termination. To our knowledge, this is the first report of effects of *sspA* on transcriptional termination.

**FIG 10:**
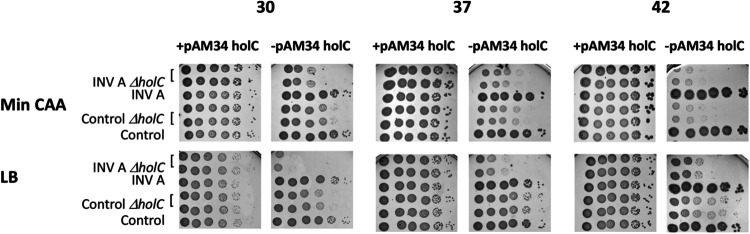
Enhancement of *holC* growth defects by a chromosomal inversion of *rrnA*, Inv A. Tenfold serial dilutions of cultures with and without the *holC* complementing plasmid (+pAM34-*holC* and −pAM34-*holC*, respectively) were plated on minimal glucose Casamino Acids (min CAA) or LB medium and incubated at 30°C, 37°C, and 42°C, as indicated. Inv A strains are plated in parallel to isogenic strains lacking the inversion (“control”). Two independent isolates were plated for the Δ*holC* derivatives.

To confirm that replication/transcription conflicts are especially problematic in *holC* mutants, we assayed *holC* effects using an E. coli strain developed by Boubakri and collaborators (51), in which a highly expressed rRNA operon has been inverted (“Inv A”) such that collisions between the transcription complex and the replication fork would be head-on. This strain carries an 18-kb inversion that includes the *rrnA* operon, which is highly expressed, increasingly so in rich medium (52, 53). The Inv A strain exhibits normal viability in minimal and LB medium, but this viability requires the function of three helicase proteins, Rep, UvrD, and DinG, that are not required for viability in noninverted strains (51). We introduced the conditional pAM34-*holC* plasmid and a chromosomal Δ*holC* into Inv A (a derivative of MG1655 [51]) and assayed plating efficiency on min CAA and LB media, at different growth temperatures (Fig. 10), with and without selection for the plasmid. The Inv A inversion exacerbated the inviability of *holC*-deficient strains, which was especially apparent on rich LB medium, where it reduced plating efficiency by several orders of magnitude (Fig. 10). Even in the presence of functional Rep, UvrD, and DinG helicases, HolC function is therefore required for full tolerance of replication/transcription collisions.

Consistent with a role of HolC in maintaining fork stability even in normal strains, inviability of *holC* mutants was exacerbated by loss of Rep helicase or double-strand break repair nuclease, RecBCD (see Fig. S2).

10.1128/mBio.00184-21.2 FIG S2 Enhancement of *holC* growth defects by loss of helicase, Rep, or recombination nuclease, RecBCD. Tenfold serial dilutions of cultures with and without the *holC* complementing plasmid (+pAM34-*holC* and −pAM34-*holC*, respectively) were plated on minimal glucose casamino acids (min CAA) and incubated at 30°C, 37°C, and 42°C, as indicated. Two independent isolates were plated for the Δ*repA* or Δ*recB* derivatives. Download FIG S2, PDF file, 0.3 MB. Copyright © 2021 Cooper et al. 2021 Cooper et al. https://creativecommons.org/licenses/by/4.0/ This content is distributed under the terms of the Creative Commons Attribution 4.0 International license.

## DISCUSSION

**FIG 11:**
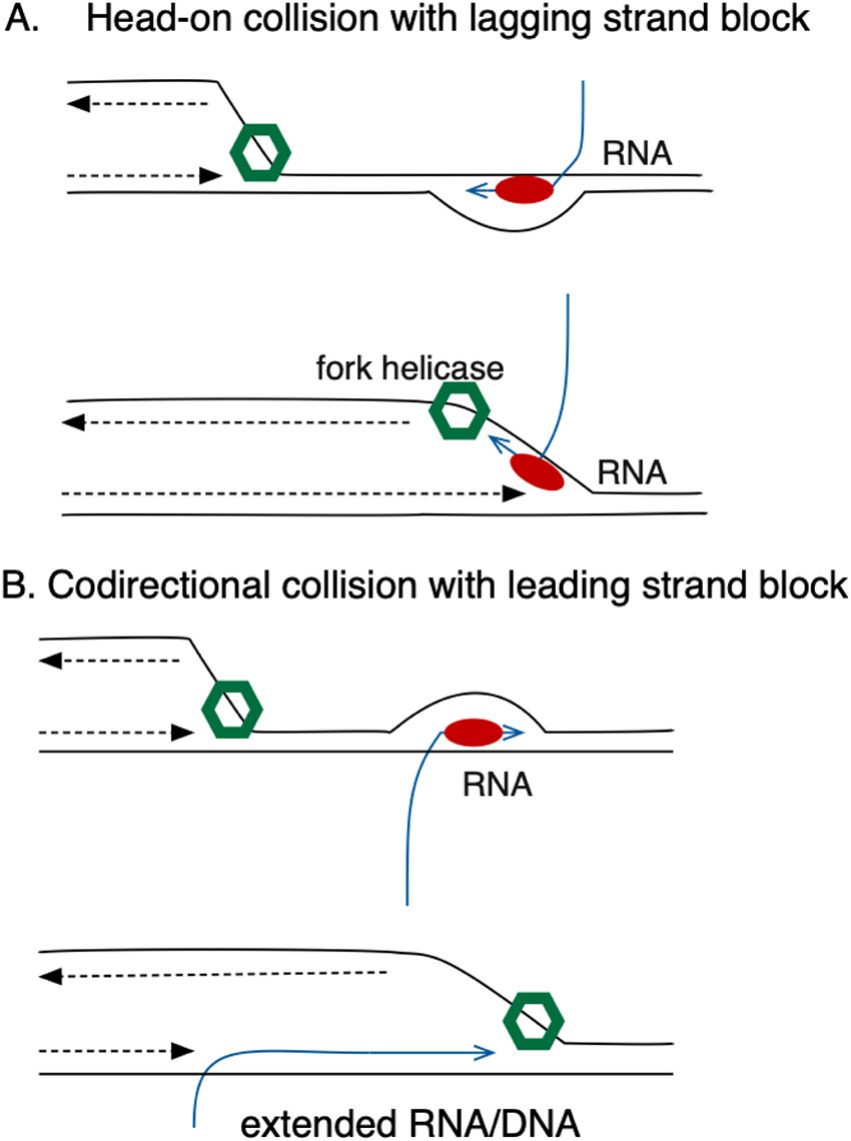
Transcription/replication conflicts. DnaB fork helicase is illustrated in green, RNA polymerase is in red, DNA is in black, and RNA is in blue. (A) Head-on collisions lead to fork arrest (B) Codirectional collisions cause uncoupling of leading and lagging strand synthesis and possible stabilization of R-loops.

Transcription elongation complexes are known to be impediments to the replication fork (reviewed in references 34, 35, and 54), and cells have evolved mechanisms to deal with these inevitable conflicts. Collisions between the replisome and transcription elongation complexes can occur in two orientations, head-on or codirectional (Fig. 11): of the two, head-on collisions are more deleterious, both *in vivo* (55–57) and *in vitro* (58–60). In bacterial genomes, gene orientation, especially for essential genes, is skewed so that most conflicts would be codirectional (61, 62). For example, in E. coli, all 7 rRNA operons are arranged codirectionally with the fork. Reversing this orientation leads to transcription stalling, increased prevalence of RNA/DNA hybrids, and requirement for helicase proteins Rep, UvrD, and DinG (51). In *Bacillus*, inversion of an *rrn* locus is even more deleterious, leading to growth impairment even in the presence of analogous helicases (63, 64).

The absence of HolC perturbs DNA replication in several ways. The suppression of *holC* by duplication of the *ssb* gene (18) suggests that replication is incomplete and the chromosome accumulates ssDNA gaps. *In vitro*, DNA replication with HolC mutants defective in SSB binding leads to uncoupling of leading and lagging strand synthesis with poor leading strand synthesis (9). The *in vivo* results presented here suggest that another function of HolC protein may be to overcome or avoid replication conflicts with transcription elongation complexes. A mutation known to reduce the stability of RNAP, RpoA R191C (31, 32), was isolated as a suppressor of the poor growth phenotype exhibited by *holC* mutants. Additional mutations in RpoA (α), RpoB (β), and RpoC (β′) subunits of RNA polymerase also acted as suppressors of *holC*; although it is unclear what biochemical defects are caused by these alleles, we think it is likely that they represent some loss of function in RNAP. Conversely, transcription factor DksA and Rho termination factor NusA sustain viability in the absence of HolC, and loss of their functions leads to synthetic growth defects with *holC*. DksA has been best studied for its role in the regulation of transcriptional initiation, where it potentiates the effects of the stringent response signaling molecule ppGpp on RNAP; *in vivo*, it is required to downregulate rRNA synthesis during amino acid starvation (42). E. coli *dksA* mutants are more prone to replication stalling and induction of the SOS response after amino acid starvation, in a manner that is reversed by inhibition of transcription with rifampicin (43). DksA is also required for replication initiated by RNA/DNA hybrids (“R-loops”) and may also assist in the removal of RNAP (65). NusA mutants are hypersensitive to bicyclomycin, an inhibitor of Rho-dependent termination, and exhibit more chromosomal fragmentation during replication (44), implicating a role for Rho-dependent termination in sustaining chromosome integrity. This work extends this finding and shows that Rho-dependent termination must be particularly critical in the absence of HolC, potentially to clear transcription elongation complexes to avoid collision with the replication fork. How HolC’s two binding partners, HolD (the clamp loader protein) and YoaA (putative helicase), participate in this role remains to be determined.

The factors required to mitigate transcription/replication collisions are complex and potentially situation specific. In E. coli, YoaA has a paralog, DinG, which is one of the DNA helicases required to survive head-on replication/transcription collisions in highly expressed genes (51). It is tempting to speculate that HolC/YoaA may aid tolerance of codirectional replication/transcription collisions, as would occur at *rrn*. Although paralogous, DinG and YoaA appear to have distinct functions: *yoaA* but not *dinG* confers sensitivity to AZT when deleted and resistance when overexpressed (27), indicating they are not merely redundant and must have specialized roles.

Because DnaB, the replication fork helicase, translocates on the lagging-strand template, a codirectional collision of the replisome with RNAP elongation complexes leads to different outcomes than a head-on collision (Fig. 11). In the codirectional orientation, DnaB can proceed unimpeded, uncoupling leading and lagging strand synthesis. The codirectional orientation can potentially lead to the use of the RNA component of an RNA/DNA hybrid (or R-loop) to reprime DNA synthesis. It has been documented that DksA aids in the use of R-loops to initiate DNA synthesis and may assist in the removal of transcription elongation complexes to facilitate repair (65, 66), which may explain how DksA sustains growth in *holC* mutants.

In addition to alleles of RNAP, we also isolated alleles of *sspA* as growth suppressors of *holC*. Mutations is *sspA* have been shown previously to suppress loss of *holD* (23). SspA, “stringent starvation protein,” is a growth-regulated RNA polymerase-associated protein (67, 68) that can act as an activator of gene expression (69). Although it is primarily expressed during the stationary phase of growth, it also regulates, either directly or indirectly, a number of genes during exponential growth (70). SspA promotes replication of bacteriophage P1 (69) as well as resistance to acid stress (71), and long-term starvation (70) and is required for virulence for many bacterial pathogens (72–78). In E. coli, it downregulates nucleoid-associated protein H-NS (71, 77). However, the suppressive effect of *sspA* on *holD* does not appear to be due to increased H-NS, since H-NS overexpression by itself does not improve the viability of HolD (23).

Whatever its mechanism, suppression of *holC* is likely to be similar to that of *holD*, but the downstream effector(s) or mechanism of SspA responsible for this suppression is currently unknown. Our observation that suppression of *holC* by *sspA* is negated in the presence of bicyclomycin suggests that it may act by affecting termination or RNAP properties, either directly or indirectly. Given that SspA is known to be a transcriptional activator, SspA may induce something deleterious to *holC* mutant strains, although it is possible that something advantageous to *holC* mutants is repressed or RNAP properties are altered in a more general way. The potential links between SspA and DNA metabolism will require further study.

## MATERIALS AND METHODS

All alleles were derived from the Keio collection (79) except as noted. All strains listed except the wild-type strains AB1157 (STL140), MG1655 (80), and PFM2 (MG1655 *rph*^+^) (81) have been transformed with the pAM34-*holC* plasmid (described below).

### Routine growth.

Bacterial cultures were routinely grown at 30°C in minimal medium plus Casamino Acids (min CAA) containing 56/2 salts, 0.2% (wt/vol) glucose, 0.001% (wt/vol) thiamine, and 0.2% (wt/vol) CAA. Plate media contained 2.0% (wt/vol) agar. For experiments testing the effects of media on growth, LB medium or minimal glucose medium were also used. LB medium contained 1% (wt/vol) tryptone, 0.5% (wt/vol) yeast extract, and 0.5% (wt/vol) NaCl with 1.5% (wt/vol) agar for plates.

### Strain construction.

The Escherichia coli strains used here were all MG1655 derivatives, except for the strains containing *nusA11*, which were AB1157 derivatives (Table 1). All alleles for mutations were derived from the Keio collection (79) except *dksA* (82), *nusA11* (83), *rpoB8* (44), *rpoC*::GFP (84), *rpoB3370* (85), and *ytfN-920*::Tn*10* (86, 87). To decrease the possibility of unintended suppressors arising, strains also contained a pAM34-*holC* plasmid which allows conditional expression of *holC*.

**TABLE 1:** E. coli K-12 strains and plasmids

Strain	Relevant genotype	Construction and/or reference
AB1157	*argE3 hisG4 thr-1 leuB6* Δ*proA62* Δ*gpt62 supE44 kdgK51 rfbD1 ara-14 lacY1 galK2 xyl-5 mtl-1 tsx-33 rpsL31* Δ*rac*	80
MG1655	*rph-1*	80
JJC3524	Δ*lacZ* Δ*attB*::*spcR*	Control strain for JJC4010; 51
JJC4010	Inv (*attL15*-*cat* attR75::*kan*)	Inv A (inv *rrnA*); 51
PFM2	*rph^+^*	81
STL22577	*rph-1*	pAM34-*holC* transformed into MG1655
STL22580	*holC*ΔFRT::*kan*	P1 *holC*ΔFRT::*kan* × STL22577
STL22677	*rpoA* dup(aa179-186) *zhd-3082*::Tn*10*	P1 *rpoA* dup(aa179-186) *zhd-3082*::Tn*10* × STL22577
STL22679	*holC*ΔFRT::*kan rpoA* dup(aa179-186) *zhd-3082*::Tn*10*	P1 *rpoA534_558* DUP *zhd-3082*::Tn*10* × STL22580
STL22715	*sspA*ΔFRT	pAM34-holC transformed to *sspA*ΔFRT
STL22717	*holC*ΔFRT::*kan sspA*ΔFRT	P1 *holC*ΔFRT::*kan* × STL22715
STL22751	*dksA*ΔFRT::*cat*	P1 *dksA*ΔFRT::*cat* × STL22577; 82
STL22753	*holC*ΔFRT::*kan dksA*ΔFRT::*cat*	P1 *dksA*ΔFRT::*cat* × STL22580
STL22757	*greB*ΔFRT	pAM34-holC transformed to *greB*ΔFRT
STL22759	*holC*ΔFRT::kan *greA*ΔFRT	P1 *holC*ΔFRT::*kan* × STL22757
STL22763	*greA*ΔFRT	pAM34-holC transformed to *greA*ΔFRT
STL22765	*holC*ΔFRT::kan *greA*ΔFRT	P1 *holC*ΔFRT::*kan* × STL22763
STL22769	*mfd*ΔFRT	pAM34-holC transformed to *mfd*ΔFRT
STL22771	*holC*ΔFRT::*kan mfd*ΔFRT	P1 *holC*ΔFRT::*kan* × STL22769
STL22960	*rpoC-gfp*	P1 *rpoC-gfp* × STL22577; 33
STL22962	*holC*Δ*FRT*::*kan rpoC-gfp*	P1 *rpoC-gfp* × STL22580
STL23028	*sulA*ΔFRT	pAM34-holC transformed to s*ulA*ΔFRT
STL23042	*holC*ΔFRT::kan *sulA*ΔFRT	P1 *holC*ΔFRT::*kan* × STL23028
STL23047	F^−^ *argE3 hisG4 thr-1 leuB6* Δ*proA62* Δ*gpt62 supE44 kdgK51 rfbD1 ara-14 lacY1 galK2 xyl-5 mtl-1 tsx-33 rpsL31* Δ*rac*	pAM34-holC transformed to AB1157
STL23049	*holC*ΔFRT::*kan*	P1 *holC*ΔFRT::*kan* × STL23047
STL23054	*nusA11 zha0132*::Tn*10*	P1 *nusA11 zha0132*::Tn*10* × STL23047; 83
STL23056	*holC*ΔFRT::*kan nusA11 zha0132*::Tn*10*	P1 *nusA11 zha0132*::Tn*10* × STL23049
STL23076	*polB*ΔFRT::*cat*	pAM34-holC transformed to *polB*ΔFRT::*cat*
STL23077	*dinB*ΔFRT	pAM34-holC transformed to *dinB*ΔFRT
STL23084	*btuB*::*Tn10 rpoB3370*	pAM34-holC transformed to *btuB*::*Tn10 rpoB3770*
STL23085	*btuB*::*Tn10 rpoB8*	pAM34-holC transformed to *btuB*::*Tn10 rpoB8*
STL23101	*holC*ΔFRT::*kan polB*ΔFRT::*cat*	P1 *holC*ΔFRT::*kan* × STL23076
STL23102	*holC*ΔFRT::*kan dinB*ΔFRT	P1 *holC*ΔFRT::*kan* × STL23077
STL23107	*holC*ΔFRT::*kan btuB*::Tn*10 rpoB3770*	P1 *holC*ΔFRT::*kan* × STL23084
STL23108	*holC*ΔFRT::*kan btuB*::Tn*10 rpoB8*	P1 *holC*ΔFRT::*kan* × STL23085
STL23348	*holC*ΔFRT::*kan ytfN-920*::Tn*10*	P1 CAG12019 × 21269; 87
STL23349	*holC*ΔFRT::*kan ytfN-920*::Tn*10*	P1 21269; 87
STL23350	*holC*ΔFRT::*kan ytfN-920*::Tn*10*	P1 *holC*ΔFRT::*kan ytfN*-*920*::Tn*10* × JJC3524 carrrying pAM34-*holC*
STL23351	*holC*ΔFRT::*kan ytfN-920*::Tn*10*	P1 *holC*ΔFRT::*kan ytfN*-*920*::Tn*10* × JJC4010 (Inv A) carrying pAM34-*holC*
STL23284	*rep*ΔFRT::*cat*	P1 *rep*ΔFRT::*cat* × STL22577
STL23285	*rep*ΔFRT::*cat holC*ΔFRT::*kan*	P1 *rep*ΔFRT::*cat* × STL22580
STL23332	*recB268*::Tn*10*	P1 *recB268*::Tn*10* × STL22577
STL23333	*recB268*::Tn*10 holC*ΔFRT::*kan*	P1 *recB268*::Tn*10* × STL22580

### Plasmid construction.

The pAM34-*holC* plasmid was constructed from pAM34 (provided by Bénédicte Michel). The XbaI-SacI fragment of pAM34 which contains the spectinomycin gene was replaced with a DNA fragment containing *holC* and its 100-bp upstream region to allow expression of *holC* from its natural promoter. Replication of this plasmid requires IPTG, which was added to between 0.15 and 0.2 mM to help maintain a low plasmid copy number and minimize deleterious effects of *holC* overexpression on cell growth.

### Growth experiments.

To test the growth of *holC* mutants, strains were grown from single colonies in the presence of 0.15 to 0.2 mM IPTG and ampicillin (Ap; 100 μg/ml) for 10 to 12 h in min CAA medium at 30°C. Cultures were then split and diluted to an *A*_590_ of approximately 0.005 in either min CAA medium containing Ap and IPTG (pAM34-*holC* maintained) or in min CAA medium alone (pAM34-*holC* lost). Growth was continued for 14 to 16 h. Next, cultures were diluted into the same media and allowed to grow to mid-late log phase (6 to 8 h), at which time they were serially diluted and plated on LB, min CAA, and min plates at 30°C, 37°C, and 42°C as indicated in the figure legends. All experiments were performed with multiple biological isolates and repeated on at least 2 days except as noted in the figure legends.

### Microscopy.

Cells depleted of pAM34-*holC* were fixed by adding equal volumes of methanol/acetic acid (3:1) to the liquid cultures. Fixed cells were then spotted onto poly-l-lysine-treated slides, washed extensively with phosphate-buffered saline (PBS), and overlaid with Vectashield mounting medium. Slides were then imaged using phase contrast with an Olympus BX51 microscope and a Qimaging Retiga 559Exi camera. The cell lengths of all of the cells in any given field of view were determined using ImageJ (88).
