# Supplementary figures and images for: The Role of Replication Clamp-Loader Protein HolC of Escherichia coli in Overcoming Replication/Transcription Conflicts
Source: mBio. 2021 Mar 9;12(2):e00184-21. doi: 10.1128/mBio.00184-21 (PMC8092217; doi:10.1128/mBio.00184-21)

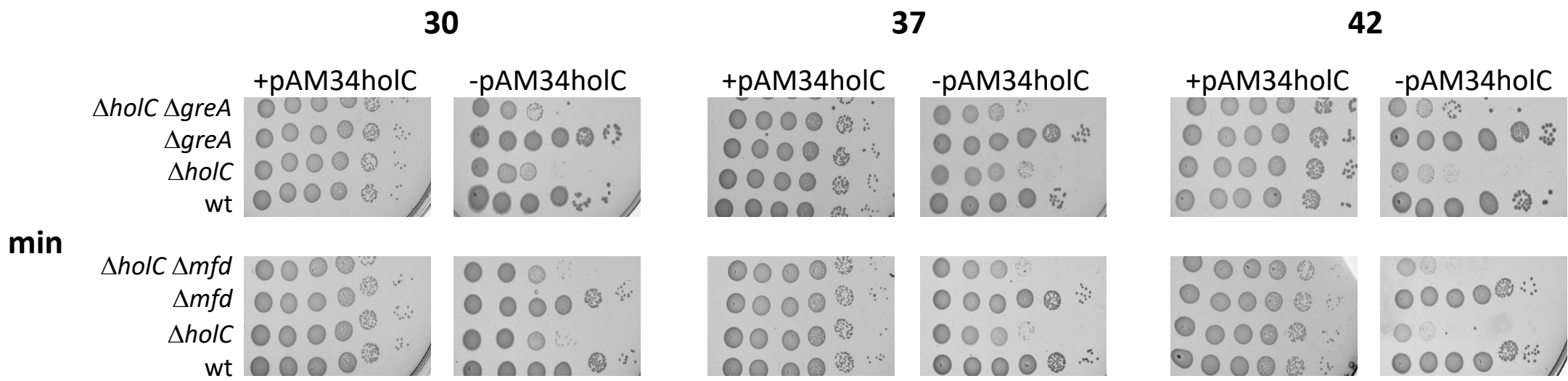

Supplement: FIG S1 [file mBio.00184-21-sf001.pdf]
