# Supplementary material for: The Role of Replication Clamp-Loader Protein HolC of Escherichia coli in Overcoming Replication/Transcription Conflicts
Source: mBio. 2021 Mar 9;12(2):e00184-21. doi: 10.1128/mBio.00184-21 (PMC8092217; doi:10.1128/mBio.00184-21)
Supplement: FIG S2 [file mBio.00184-21-sf002.pdf]

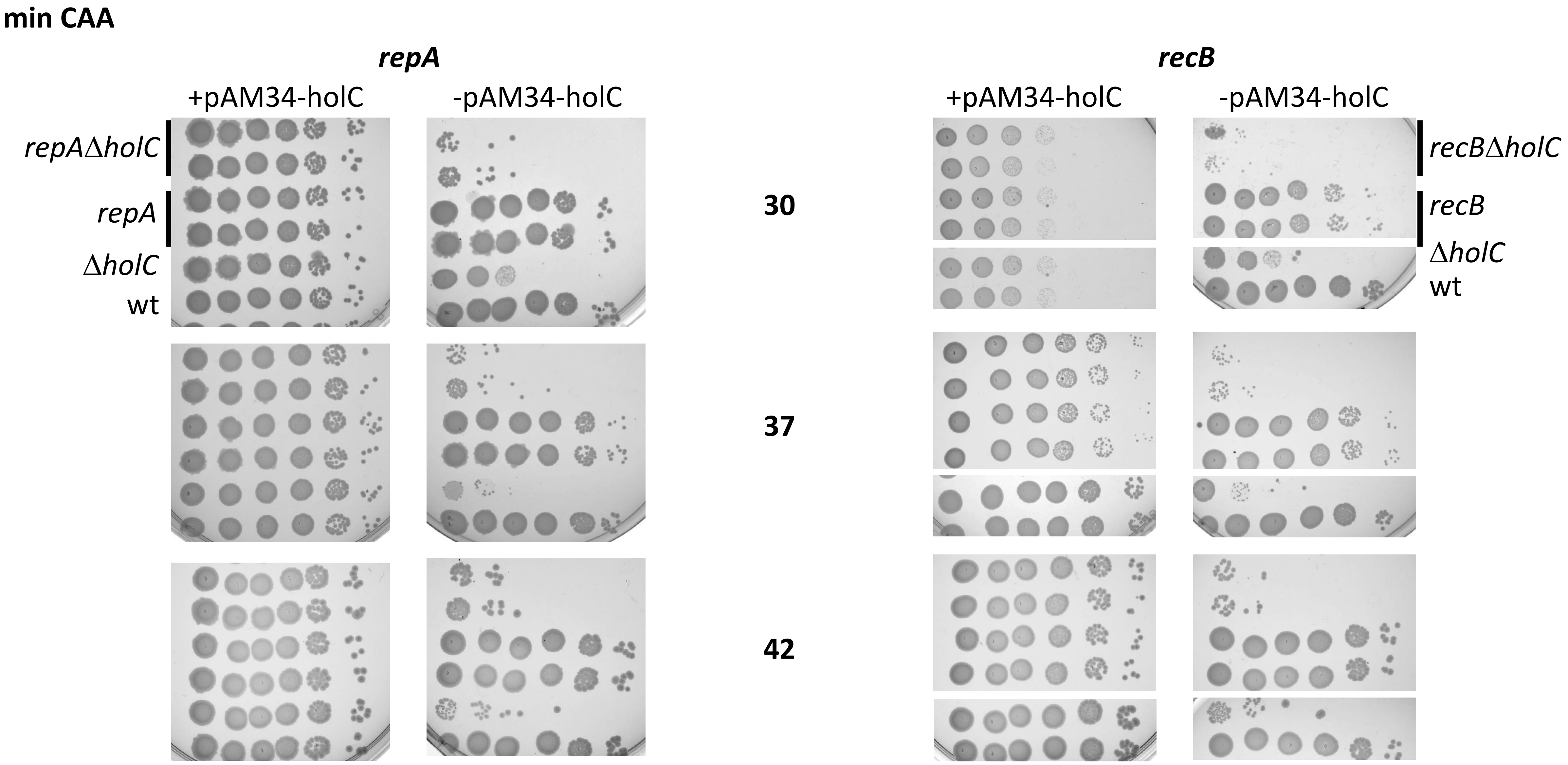

min CAA
repA
recB
+pAM34-holC
-pAM34-holC
+pAM34-holC
-pAM34-holC
repAΔholC
repA
ΔholC
wt
30
37
42
recBΔholC
recB
ΔholC
wt
